# Intermittent Pressure.—Its Relation to Orthodontia

**Published:** 1888-04

**Authors:** Levitt E. Custer

**Affiliations:** Dayton, O.


### Intermittent Pressure. Its Relation to Orthodontia.

BY LEVITT E. CUSTER, B.S., D.D.S., DAYTON, O.

Read Before the Mississippi Valley Dental Association.

No branch of our esteemed profession has received such an impetus of late as that of correcting irregularities of the natural teeth. In fact, it has reached such a point that the child has received a scientific name—they call it Orthodontia—and its devotees are wrangling over the priority of invention.

To move teeth in the alveolus we are dependent upon the same fundamental principle of the animal economy as that by which all change of shape in osseous structures is accomplished, namely; resorbtion and deposition. The theory was once held that there was an interstitial change, but at present we believe there is an actual digestion and excavation in one portion, and a new deposition taking place in another. During the preparation of the lower jaw for the permanent molars the ramus is carried back, not by an interstitial development, but by a resorption anteriorly, and a deposition posteriorly. When lime-salts have once been deposited as the basis substance of bone, they, by virtue of their intense hardness and heterogeneous nature, retain that form until reduced by retrograde metamorphosis to the embryonic condition.

Resorbtion, as a physiological process, takes place under the agency of a class of cells which may be understood to have a retrograde function. They break down tissue already built, and under accidental conditions reduce foreign substances of animal origin to a condition fit for assimilation by osmosis. Such cells, according to where found, have been termed leucocytes, giant cells, osteoclasts or odontoclasts.

The above cells, even though of retrograde function, like all others are brought into action by adequate stimuli. Every different cell requires a special excitation; for the cells of the salivary glands to act, there is reflex nervous irritation originating in the gustatory cells; for muscle cells, motor impulse; for giant cells, a thrombus, an infarct or foreign body; for odontoclasts or osteoclasts, the effort of nature to change the size or shape of a pre-existing osseous structure. Osteoclasts are also stimulated by pressure, and upon the latter do we depend for change of shape in the alveolar process for correcting irregularities. When nature has once fashioned the alveolus and the arrangement of the teeth to satisfy her own typal demands, we can ask no more of her as a stimulant but must resort to the other method; namely, pressure. All the forms of pressure for regulating resolve themselves into either of two classes of forces, intermittent or constant. Under the former may be classed those forces which move to a definite distance and there remain stationary, such as the screw in all its forms: under the latter, constant force, all those which bear with an elastic or continued pressure, such as the spring or rubber bands. Intermittent pressure acts intermittently as such because the space gained by absorbtion is not followed up immediately by new pressure only as it is applied by the operator.

The method by which giant cells, leucocytes, osteoclasts or odontoclasts, cells of retrograde function, reduce substances has been designated by Dr. Black as resorptive digestion. We understand these cells to have the property of throwing out a digestive fluid which acts as a soluble ferment analogous to that of ptyaline, trypsin or pepsin. In each case this fluid varies according to the nature of the object to be digested. Krause maintains that in absorbtion of osseous tissue this fluid is lactic acid. In order to act these cells arrange themselves so as to be in direct apposition with the part to be digested, they come in actual contact. Implanted teeth, the roots of which are undergoing absorption, contain pits which are lined with odontoclasts. In the formation of sequestra of bone, osteoclasts are found at the line of union of the dead with the living; ligatures are surrounded and crowded with leucocytes. In sponge-graft granulations are filling up the pores while giant cells are preparing the walls for osmosis, and blood-clots become organized by the presence of connective tissue cells. Dr. Black says, "The osteoclasts are not attached to the surface of the bone or tooth by any mechanical means whatever; they simply lie against the surface and are detached with the least movement. They only act, however, when lying in contact with the surface. Any intervening substance whatever will prevent their action."

From the foregoing it is evident that the action of osteoclasts may be lessened or entirely suspended by either of two conditions; dilution of the digestive fluid, or separation of the cells from the point of absorption. When resorption is produced artificially, and it is agreed that pressure is necessary to stimulate osteoclasts, as the result of pressure there are associated conditions effecting the action of these cells, which vary according to the method used. By one method of pressure the digestive fluid may be weakened, or by another the cells may be separated from the seat of absorption by intervening bodies. Any form of pressure in orthodontia, if continued long enough and hard enough, will produce hyperæmia followed by inflammation and possibly by suppuration or abscess. When hyperæmia occurs we have a serous and a corpuscular exudate which infiltrate the surrounding tissue. Serum being a liquid which in small quantities is taken up by the lymphatics, but when increased, producing

œdema, readily comes in contact with the digestive fluid of the osteoclasts which is diluted and resortpion retarded. On the other hand the blood corpuscles of hyperæmia not being liquid insinuate themselves between the cells and the point of absorption and become a mechanical obstruction to their action. In inflammation we have as well as the serous and corpuscular an additional one, fibrinous, which at first acts mechanically and after liquefaction becomes a menstruum for weakening the digestive fluid. The result of mechanical obstruction to the action of these cells is no better shown than by the resorption of the roots of temporary teeth where the process is stopped by the occurrence of abscess. In this instance the resorptive cells become disseminated among the pus corpuscles and not being able to act unless in contact, the process ceases. Resorption is more marked when the digestive fluid is undiluted, but when it is disseminated in the serous exudate of hyperæmia, fibrinous exudate of inflammation, or the pus corpuscles of abscess, it becomes retarded. Herein lies the problem of the efficiency of the different methods—to stimulate retrograde cellular activity without producing excessive hyperæmia or inflammation.

Cognizant of the fact that resorption produced by artificial means or by external agents is not to be compared with natures physiological process in that we have to deal with two opposites—to stimulate osteoclasts by pressure and at the same time prevent excessive hyperæmia and inflammation, the results of pressure—we may yet by a peculiar method of pressure produce a process approaching very closely natures own. Such a method of pressure besides being sufficient to stimulate osteoclasts to action shall allow, first, regain of tone to the blood-vessels and lymphatics in the region of pressure; and second, it shall allow free exchange of pabulum and rapid entrance into the lymphatics of the dissolved bone tissue. Of the first condition, I say there shall be regain of tone in the vessels, because pressure of any kind tends to paralysize the vaso-motor nerves in that region and hyperæmia will result. Let the pressure be continued and the exudates of hyperæmia will follow, which will retard the action of the osteoclasts in the two ways that have been shown. On the other hand let there be an intermittent pressure and the room gained by the osteoclasts while under pressure will allow regain of tone to the vessels during that period which some have denominated " rest."

The osteoclasts like all other cells has three properties, ingesta, ossimilation, and excreta ; it also ceases to act when this excreta is not removed. Since the lymphatics under pressure cannot so readily absorb the waste products of the body, and since under continued pressure we have continued stimulation and continued action of osteoclasts, as a result, we have a continued formation of excreta in the most unfavorable conditions for its removal : but let this pressure have been such that will expand to a definite distance and there remain stationary, then the space gained by active work of tbe osteoclasts before their action is stopped by the excreta or dissolved lime-salts will allow their escape by the lymphatics which have regained tone. Under continued pressure there is not a free exchange of fluids ; mechanical resistance keeps back pabulum, and the osteoclasts become surrounded by their own debris.

Besides the peculiar fitness and adaption of intermittent pressure in the above two fundamental conditions for a physiological performance of resorbtion, there are other minor qualities which render it still more desirable as a system of pressure to be used in orthodontia. More force may be applied by this method than by the other, and the teeth acting as a lever will under this increased force produce enough pressure, not on the edge alone, but in the body of the alveolus to stimulate osteoclasts in that portion. Were elastic pressure applied even to inflammation this would not be strong enough to produce resorbtion at any other point than directly opposite the tooth. I think there is more in this than we are willing to admit. To produce any other resorbtion than opposite the tooth by elastic force the peridental membrane would run high in inflammation. This is an easy thing to accomplish with intermittent force since we are able to apply many times the amount of pressure with less danger of inflammation.

It is a *positive* method and distance gained by its use is definite ; it may be measured with mathematical precision ; also holds what has been gained.

It needs no renewing, once in place it may be used as a general thing, until the end is accomplished. As a result the new depositions of osseous materials are not interfered with as they would be when the tooth is drawn back by the fibers of the peridental membrane during change of apparati: the death of pulps has been attributed to breaking or changing of appliances.

Under intermittent pressure we have a work almost painlessly performed, because more nearly a physiological process. According as are all either constructive or retrograde processes physiologically performed are they painless and as they approach the pathological do they become painful. We have a work more progressive, because of continued action under natural laws,—not an action that is half inflammatory; and a work that is more effective, because produced by causes which work more in harmony with nature; which allows regain of tone, free exchange of pabulum, and more favorable conditions for contact of resorber with resorbed and an undiluted action of the digestive fluid.

DISCUSSION.

*Dr. Watt:* I think I was among the first to advance this thought. I see Dr. Angle says to " keep up the pressure without intermission." Another place he says " there should be no back sliding." I think Dr. Angle meant to convey the idea that the pressure should not be lessened enough to let the tooth take a retrograde movement. This should be avoided; but the pressure should be released enough occasionally to give the tooth and associate parts a rest. When a tooth is moved backward or forward, a portion of the peridental membrane is destroyed. The same thing occurs when rotating, only it is more severe; and unless you give the tooth an occasional rest, it is liable to cause severe inflammation.

*Dr. H. A. Smith* agreed with the paper and thought it according to physiological laws; and yet, good results had been brought about by continued pressure. He believed that in the continued pressure method the teeth receive more rest than is generally supposed.

*Dr. Wright* said he liked to see a mixing up of physiology with mechanics. That is the plan on which we ought to operate in for it raises us up above the common level.

